# Treatment Efficiency in Gaucher Patients Can Reliably Be Monitored by Quantification of Lyso-Gb1 Concentrations in Dried Blood Spots

**DOI:** 10.3390/ijms21134577

**Published:** 2020-06-27

**Authors:** Claudia Cozma, Paskal Cullufi, Guido Kramp, Marina Hovakimyan, Virtut Velmishi, Agim Gjikopulli, Sonila Tomori, Steffen Fischer, Sebastian Oppermann, Ulrike Grittner, Peter Bauer, Christian Beetz, Arndt Rolfs

**Affiliations:** 1 CENTOGENE AG, Am Strande 7, 18055 Rostock, Germany; Claudia.Cozma@centogene.com (C.C.); Guido.Kramp@centogene.com (G.K.); Marina.Hovakimyan@centogene.com (M.H.); Steffen.Fischer@centogene.com (S.F.); Sebastian.Oppermann@centogene.com (S.O.); Ulrike.Grittner@charite.de (U.G.); Peter.Bauer@centogene.com (P.B.); Arndt.Rolfs@centogene.com (A.R.); 2 Pediatric Department, University Hospital ’Mother Teresa’, 1000 Tirana, Albania; PaskalCullufi@gmail.com (P.C.); tutimodh@yahoo.com (V.V.); agimgjikopulli21@gmail.com (A.G.); s_tomorius@yahoo.com (S.T.); 3 Institute of Biometry and Clinical Epidemiology, Charité—Universitätsmedizin Berlin, Charitéplatz 1, 10117 Berlin, Germany; 4 Berlin Institute of Health (BIH), Anna-Louisa-Karsch-Str. 2, 10178 Berlin, Germany

**Keywords:** biomarker, enzyme replacement therapy, Gaucher disease, Lyso-Gb1, monitoring

## Abstract

Gaucher disease (GD) is a lysosomal storage disorder that responds well to enzyme replacement therapy (ERT). Certain laboratory parameters, including blood concentration of glucosylsphingosine (Lyso-Gb1), the lyso-derivate of the common glycolipid glucocerebroside, correlate with clinical improvement and are therefore considered candidate-monitoring biomarkers. Whether they can indicate a reduction or loss of treatment efficiency, however, has not been systematically addressed for obvious reasons. We established and validated measurement of Lyso-Gb1 from dried blood spots (DBSs) by mass spectrometry. We then characterized the assay’s longitudinal performance in 19 stably ERT-treated GD patients by dense monitoring over a 3-year period. The observed level of fluctuation was accounted for in the subsequent development of a unifying data normalization concept. The resulting approach was eventually applied to data from Lyso-Gb1 measurements after an involuntary treatment break for all 19 patients. It enabled separation of the “under treatment” versus “not under treatment” conditions with high sensitivity and specificity. We conclude that Lyso-Gb1 determination from DBSs indicates treatment issues already at an early stage before clinical consequences arise. In addition to its previously shown diagnostic utility, Lyso-Gb1 thereby qualifies as a monitoring biomarker in GD patients.

## 1. Introduction

Gaucher disease (GD) is an autosomal recessive disorder caused by biallelic variants in the *GBA* gene [1]. *GBA* encodes beta-glucocerebrosidase, a lysosomal hydrolase. Absent or reduced activity of this enzyme results in accumulation of its substrate glucosylceramide (GlcCer). Macrophages that contain large amounts of lysosomal GlcCer (“Gaucher cells”) are found in several tissues of GD patients and are considered the major mediators of pathology. The primary phenotypic consequences are organomegaly (liver and spleen), hematological abnormalities, and bone disease; some patients also develop neurological symptoms. Treatment options include substrate reduction therapy (SRT) and enzyme replacement therapy (ERT) [2]. In the majority of patients, clinical as well as laboratory parameters significantly improve under therapy [3].

While the impairment of beta-glucocerebrosidase in GD results in accumulation of its primary substrate, GlcCer, a number of additional lipids have been found to be enriched in GD patients. One of these is glucosylsphingosine (also referred to as GlcSph, Lyso-GL1, or Lyso-Gb1). This deacylated form of GlcCer was first isolated from adult GD patients’ spleens [4] and brains [5]. It was subsequently also found in other tissues, including those from affected fetuses and mice [6]. The hypothesis that Lyso-Gb1 is of pathogenic/toxic relevance for GD [5] received strong recent support as a result of observations of mice injected with this metabolite [7].

With the availability of methods for reliable quantification in plasma, Lyso-Gb1 was revealed to be an excellent diagnostic biomarker for GD, outperforming other markers such as chitotriosidase enzymatic activity and CCLC18 [8,9]. Confirming preliminary observations of these initial studies, Lyso-Gb1 levels were subsequently shown to decrease in response to treatment [10,11,12,13,14]. By additionally considering that untreated patients with a mild form of the disease have stably low Lyso-Gb1 levels [15], therapeutic monitoring of GD patients based on Lyso-Gb1 appears highly advisable. While Lyso-Gb1 has usually been determined from plasma (e.g., [8,12]), quantification from dried blood spots would provide several advantages. These include (1) highly reduced sample volume requirements, (2) less impact of shipment and storage conditions, (3) easier transportation including from remote areas, and (4) an opportunity for reliable self-sampling [16]. Despite application by a few recent studies (e.g., [14,15]), a proper validation of this approach has been lacking.

For obvious ethical reasons, the effects of discontinuing treatment in GD patients have not been analyzed in the frame of well-designed studies. Based on a small series of 15 patients who withdrew from ERT, mainly for personal concerns or because of financial constraints, Elstein and colleagues concluded that temporary “treatment vacation” is worth consideration under certain circumstances [17] and thereby triggered a lively debate (e.g., [3,18]). A more extensive set of data became available due to a global shortage of the recombinant enzyme imiglucerase (Cerezyme) in 2009/2010 [19]. Several studies subsequently reported consequences of the resulting treatment breaks and/or dose reductions. Laboratory parameters responded early, and most affected patients also showed a continuous clinical deterioration (e.g., [20,21,22]). Treatment interruptions are thus not considered for the management of GD patients any longer [23]. Proper evaluations of how and when inefficiency/absence of a therapeutic effect can be picked up by newly available biomarkers are therefore not usually feasible.

The present study validates dried blood spot (DBS)-based quantification of Lyso-Gb1 and analyzes longitudinal performance of the assay in stably treated GD patients. It also harnesses unusual circumstances that resulted in a forced break in ERT to show that Lyso-Gb1 reliably flags the loss of a therapeutic effect. It thereby establishes DBS-based Lyso-Gb1 quantification as a feasible and highly valid strategy for therapeutic monitoring of GD patients.

## 2. Results

### 2.1. Lyso-Gb1 Quantification in Dried Blood Spots Is a Valid Laboratory Approach Including Excellent Precision Characteristics

DBS-based quantification of Lyso-Gb1 was formally validated in the context of applying for accreditation from Clinical Laboratory Improvement Amendments (CLIA) and the College of American Pathologists (CAP). Table 1 details precision as the parameter that is probably the most critical for a monitoring test. We found coefficients of variation (CVs) for intra-assay precision to be <6% across the whole range of relevant concentrations. Moreover, sample age in a relevant time interval had no impact on Lyso-Gb1 quantification (Table 1). We concluded that the assay is valid and able to precisely determine Lyso-Gb1 concentrations in DBS samples.

### 2.2. Under Continuous Treatment, Lyso-Gb1 Levels Vary over Time in a Patient-Specific Manner

**Figure 1:**
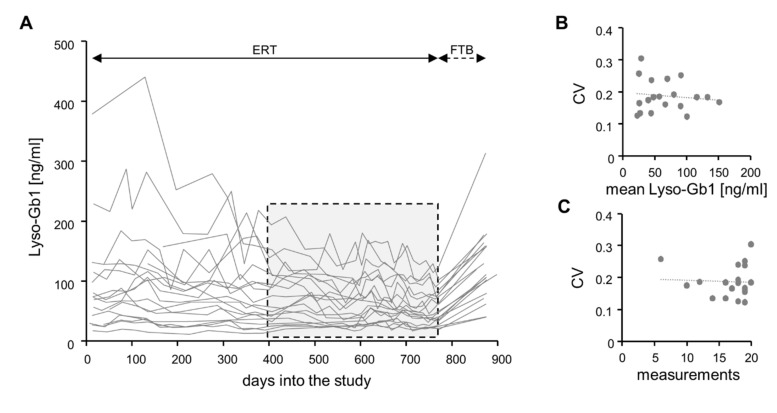
Lyso-Gb1 values in a cohort of 19 routinely monitored Gaucher patients. (**A**) A total of 517 measurements are depicted as one line per patient. The stippled box denotes the subset of 317 measurements over a ~12-month period on which the diagrams in Figure 1B,C, and Figure 2 are based (compare also text). (**B**) Patient-specific coefficients of variation are plotted against patient-specific mean Lyso-Gb1 values. (**C**) Patient-specific coefficients of variation are plotted against the number of measurements available per patient. CV, coefficient of variation; ERT, enzyme replacement therapy; FTB, forced treatment break; trendlines were set to linear.

We next analyzed the Lyso-Gb1 values obtained for the 19 patients in our study over time. During the ~25-month period of continuous ERT, Lyso-Gb1 values showed a general tendency to decrease. This tendency was more pronounced in some patients and less pronounced in others but seemed to become weaker over time (Figure 1A). We therefore focused our subsequent analyses on the last 12 months of the period in question (stippled rectangle in Figure 1A).

A total of 317 measurements were collected in this period. This corresponds to a mean of 16.7 measurements per patient (median 18; range 6 to 20). Individual Lyso-Gb1 values differed by more than one order of magnitude (minimum 16.6 ng/mL; maximum 207 ng/mL), while patient-specific mean values differed up to 7-fold (minimum 22.3 ng/mL; maximum 151.0 ng/mL). For all patients, CVs were greater than those determined for precision; they ranged from 12% to 30% (median 18%). As CVs neither correlated with absolute Lyso-Gb1 levels (Figure 1B) nor with measurement frequency (Figure 1C), the extent of temporal fluctuation seemed to be a patient-specific parameter.

### 2.3. Temporal Fluctuation Is Leveled Out by Frequent Measurements over Long Reference Intervals

**Figure 2:**
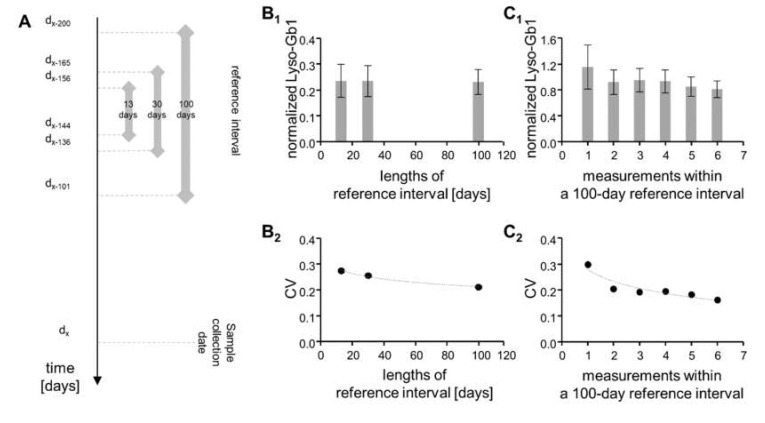
Normalization of Lyso-Gb1 based on the data highlighted in Figure 1A. (**A**) Scheme that depicts the tested normalization strategies. The most recent value (dating from day d_x_) is divided by the mean value as calculated from the three indicated reference intervals. (**B**) Mean normalized Lyso-Gb1 (**B_1_**) and corresponding coefficients of variation (**B_2_**) of the normalized values are provided for each of the three reference intervals. (**C**) Mean normalized Lyso-Gb1 (**C_1_**) and corresponding coefficients of variation (**C_2_**) of the normalized values within the 100-day reference interval are plotted against the number of underlying measurements. CV, coefficient of variation; trendlines were set to logarithmic.

Monitoring a patient may be based on comparing (normalizing) the current value against a single previous reference value. Given the extent of variation observed above, we considered using means from reference intervals as an additional strategy for normalization. A large reference interval was set to 100 days (harboring up to eight values in case of uninterrupted sample collection every 14 days), while a small reference interval was set to 30 days (containing up to three values). A 13-day interval was considered to guarantee presence of not more than one value. The 30-day and 13-day intervals were placed centrally to the 100-day interval, which covered days −200 to −101 (Figure 2A).

The below calculations are based on the 1-year period marked by the stippled square in Figure 1A.

For all three intervals, the values at the time of sample collection were slightly lower than the corresponding reference values (between 92% and 94% of the reference mean). For the 13-day interval, the corresponding CV was 27.4%, while it dropped to 25.5% for the 30-day interval and further to 21.0% for the 100-day interval (Figure 2B). This is consistent with a longer reference interval leveling out some of the variation. As a next step, we analyzed whether a similar effect was conferred by the frequency of monitoring. We indeed observed that higher numbers of values within a 100-day period correlated with lower CVs (Figure 2C). Collectively, these observations argue in favor of basing monitoring efforts on the combination of long reference intervals and frequent measuring.

### 2.4. Normalized Lyso-Gb1 Values Are Highly Sensitive and Specific for Flagging Absent Treatment

**Figure 3:**
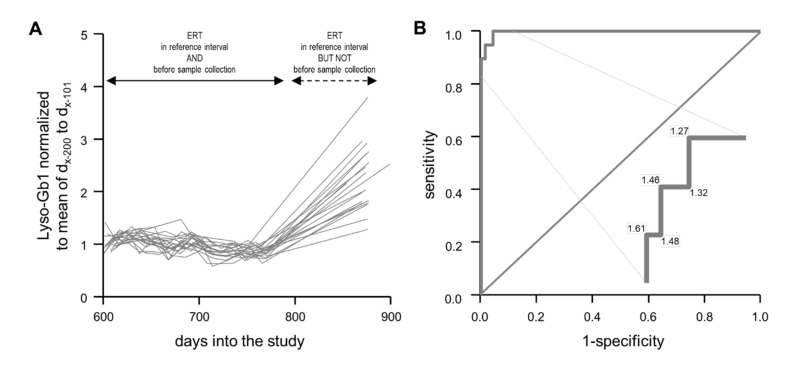
Effect of a treatment break on Lyso-Gb1 values as normalized to the 100-day reference interval (compare text and Figure 2). (**A**) The 227 relevant values are depicted as one line per patient. (**B**) Receiver operating characteristic curve for discriminating “treatment break” values from the “continuous treatment” values. The enlarged segment of the curve denotes normalized Lyso-Gb1 values corresponding to changes in sensitivity and specificity. ERT, enzyme replacement therapy.

Having defined frequent measurements within 100-day reference intervals as appropriate for capturing Lyso-Gb1 kinetics in periods of stable treatment, we analyzed the treatment break data with this approach. To this end, we plotted all relevant data (Figure 3A). The normalized Lyso-Gb1 values were around 1.0 for all patients when they received ERT. They did, in fact, even show a tendency for becoming ever lower over time. In stark contrast, the normalized values had increased to between 1.27 and 3.79 (median 2.16) after the forced treatment break. In combination, these data enable discrimination of “under treatment” versus “not under treatment” with high sensitivity and specificity (Figure 3B). The range of partial overlap (1.27 to 1.61) is small and accounts for less than 5% of all values (11 of 228).

## 3. Discussion

The present study is conceptually bipartite. In its first part, it defines the longitudinal performance of Lyso-Gb1 determination from DBSs in GD patients that are under continuous ERT. The underlying data set is unprecedented in several ways. First, it focuses on measurements at times of ongoing therapy, while related previous studies usually compared pretreatment states with post-treatment states (e.g., [12,13]). Second, it contains an average of 27 independent data points per patient (517 measurements for 19 patients), while in the largest of the previous studies [13,24], this figure was considerably lower. Finally, almost all of the consecutive measurements were less than 3 months apart, while 3 months was the usual minimum interval previously [8,9,10,12,13,24]. This high density of the data set is a key novelty of our study. It reveals considerable fluctuation that strongly exceeds precision (compare Figure 1 and Table 1). The extent of this fluctuation appeared comparable to what others observed over longer intervals in both DBSs (e.g., [24]) and plasma (e.g., [13]), though no specific calculations had been provided there. Collectively, these findings argue that variation is a real phenomenon. Potential explanations include (1) intercurrent disease processes, (2) impact of the circadian rhythm, and (3) effects of nutrition and/or physical activity. Notably, period times for fluctuation seem to be in the range of days or even shorter. Along this line, we note a statement by Smid and coworkers, who mention an “unexpected transient increase” of Lyso-Gb1 in samples taken 4 h after first treatment [10]. Further studies that monitor more frequently are clearly needed. Independent from the causes and period times of variation, the corresponding observations need to be accounted for in the development of monitoring strategies. We succeeded in leveling out a fraction of the variation by applying a long reference interval instead of a single reference value (Figure 2B). In addition, we found that low variation was associated with small measurement intervals (Figure 2C). Maybe not surprisingly, these observations suggest that monitoring is most efficient when regularly and densely done over a long period of time.

The second part of our study builds on an unforeseen discontinuation of treatment. Such “treatment holidays” have been known from case reports (e.g., [18,25,26]) and from a small heterogeneous patient series [17] for almost 20 years. The 2009/2010 shortage of imiglucerase [19] provided more extensive corresponding data (e.g., [20,21,22]). Generally, the referenced studies observed a worsening of first the laboratory and then the clinical parameters. Similar findings, though less serious, were reported as a result of a reduction in ERT dosage [22,27] and a prolongation of treatment intervals [28]. Probably due to the relative shortness of the break, patients in the current study did not report a worsening of GD-related symptoms. Lyso-Gb1, however, increased in all patients (Figure 1A and Figure 3A). As this increase was much larger than the usual variation, untreated and treated conditions could be discriminated between with almost 100% sensitivity and specificity (Figure 3B). This newly recognized ability of Lyso-Gb1 to indicate treatment inefficiency parallels its rapid response to initiation of treatment (e.g., [13]) and improvement of treatment [12]. Though chitotriosidase activity and CC chemokine ligand 18 (CCL18), i.e., alternative monitoring parameters [29], may be of similar value in certain patients, they are less specific [30,31], not involved in the disease’s pathophysiology [32], and/or not applicable to all individuals [33].

## 4. Materials and Methods

### 4.1. Patients

The present study was based on a cohort of 19 mainly unrelated Gaucher patients for whom Lyso-Gb1 had regularly been monitored over up to 40 months. It was approved by the Albanian Ethic Committee (Protocol Number 095, Tirana (7th July 2015)). As monitoring of the first patients started in November 2016, we defined November 1 of this year as day 1 of the study in all analyses. While monitoring of all patients is ongoing, the end of the forced treatment break (see below for details) was arbitrarily defined as the endpoint for the data reported in the present publication.

All patients were followed at a single center in Albania by the same team of expert physicians. Twelve of the patients were male, and seven were female. Their ages at the start of the study ranged from 6 to 78 years (median 22 years). They all manifested with typical symptoms of the disease and had received a definite genetic diagnosis based on identification of biallelic pathogenic or likely pathogenic variants in the *GBA* gene. With 13 occurrences among the 19 patients (68%), the predominant genotype was compound heterozygosity for c.1226A > G (p.(N409S), i.e., one of the most frequent Gaucher-associated *GBA* variants [34]) and c.[882T > G;1342G > C] (p.([H294Q;D448H]), the so-called “Balkan allele” [35]). Details as to phenotypes and genotypes will be reported as part of a larger clinical-genetic publication elsewhere (manuscript submitted). All patients were under a largely unchanged ERT treatment regime until mid-December 2018, corresponding to ~day 780 of the study (taliglucerase alfa; 45–60 units per kg body weight; administration every 14 days); the exceptions constituted single, slight dose reductions 150 to 200 days into the study in eight patients. Between December 2018 and March 2019, treatment had to be interrupted for 90–100 days based on unavailability of ERT due to temporal administrative issues.

### 4.2. Quantification of Lyso-Gb1

DBS samples (CentoCard) were prepared from ethylendiamine tetraacetic acid (EDTA) blood drawn just before administration of the enzyme. From one DBS, three punches of diameter 3.2 mm were obtained using a DBS puncher (Perkin Elmer, Waltham, MA, USA), plated in 2.2 mL round-bottom tubes (Eppendorf AG, Hamburg, Germany), and layered with 50 μL extraction solution (DMSO:ddH_2_O, 1:1) and 100 μL internal standard solution. Samples were then mixed using a multitube vortex device at 2500 rpm for 30 s, kept at 37 °C for 30 min under agitation at 700 rpm, and sonicated for 1 min at maximum power. Two hundred microliters of the sonicated sample were transferred to an AcroPrep filter plate with polytetrafluoroethylene (PTFE) membrane (Pall Corporation, Port Washington, NY, USA) placed on a 96-well, V-shaped-bottom plate (VWR International, Radnor, PA, USA). Any solid particles were removed by filtration (centrifugation for 5 min at 3500 rpm in a Hermle Z300 plate centrifuge (Hermle Labortechnik, Wehingen, Germany)).

Mass spectrometric analysis was performed using a Waters ACQUITY UPLC (Waters Corporation, Milford, MA, USA) coupled with an ABSciex 5500 Triple Quad mass spectrometer (Sciex, Framingham, MA, USA). The following transitions were monitored in the mass spectrometric analysis: 624.4→282.2 for the internal standard and 464.4→282.2 for Lyso-Gb1. A water-based calibration curve was added to each batch for all analyses performed for this study, with concentration ranging from 0 to 1000 ng/mL. For each batch, a quality check utilized samples with known Lyso-Gb1 concentrations (Gaucher patients and healthy controls); the analyses were considered valid only when all these controls were in the predetermined range.

Validation of Lyso-Gb1 quantification from DBSs followed International Organization for Standardization (ISO) 15189 [36].

## 5. Conclusions

The present study validates Lyso-Gb1 quantification from DBSs. It also reveals the extent of variation of this biomarker over comparatively short time intervals and introduces concepts for how to address the resulting challenges. It eventually reveals that monitoring of Lyso-Gb1 enables flagging of absent/insufficient treatment before clinical consequences arise. With DBSs facilitating self-sampling [37] and with handheld mass-spectrometry devices on the horizon [38], determination of Lyso-Gb1 from filter cards thus appears an ideally suited monitoring tool for the clinical trials expected to be launched in the near future.

## Figures and Tables

**Table 1:** Precision for determination of glucosylsphingosine (Lyso-Gb1) from dried blood spot (DBS) samples.

		Control Sample	Control Sample Spiked with Low Levels of Lyso-Gb1	Control Sample Spiked with High Levels of Lyso-Gb1
Fresh sample	Mean ^a^ [ng/µL]	5.3	29.7	217.2
	SD [ng/µL]	0.3	1.8	10.6
	CV [%]	4.8	5.9	4.9
After 3 months of storage	Mean ^a^ [ng/µL]	5.6	27.0	218.5
	SD [ng/µL]	0.2	1.4	10.2
	CV [%]	3.1	5.3	4.7

^a^ Based on analyzing DBS punches from 10 control individuals each; SD, standard deviation; CV, coefficient of variation.
